# MiR-122 Directly Inhibits Human Papillomavirus E6 Gene and Enhances Interferon Signaling through Blocking Suppressor of Cytokine Signaling 1 in SiHa Cells

**DOI:** 10.1371/journal.pone.0108410

**Published:** 2014-09-29

**Authors:** Junming He, Yuting Ji, Aimei Li, Qingmeng Zhang, Wuqi Song, Yujun Li, Hongxin Huang, Jun Qian, Aixia Zhai, Xin Yu, Jinyun Zhao, Qinglong Shang, Lanlan Wei, Fengmin Zhang

**Affiliations:** 1 Department of Microbiology, Harbin Medical University, Harbin, Heilongjiang, China; 2 The Heilongjiang Key Laboratory of Immunity and Infection, Harbin, Heilongjiang, China; 3 The Key Laboratory of Pathogenic Biology, Heilongjiang Higher Education Instructions, Harbin, Heilongjiang, China; 4 IBP-UC Group for Immunotherapy, CAS Key Laboratory for Infection and Immunity, Institute of Biophysics, Chinese Academy of Sciences, Beijing, China; Karolinska Institutet, Sweden

## Abstract

Human Papillomavirus (HPV) 16 infection is considered as one of the significant causes of human cervical cancer. The expression of the viral oncogenes like E6 and E7 play an important role in the development of the cancer. MiR-122 has been reported to exhibit a strong relationship with hepatitis viruses and take part in several tumor development, while the effects of miR-122 on HPV infection and the HPV viral oncogenes expression still remain unexplored. In this study, using RNAhybrid software, the potential binding sites between miR-122 and HPV16 E6 and E7 mRNAs were identified. Over and loss of miR-122 function showed that miR-122 could directly bind with HPV16 E6 mRNA and significantly inhibit its expression in SiHa cells, which was further confirmed by constructing the miR-122-E6-mu to eliminate the miR-122 binding effects with E6. The increase of the expression of type I interferon (IFN) and its classical effective molecules and the phosphorylation of signal transducers and activators of transcription (STAT1) protein indicated that miR-122 might enhance type I interferon in cervical carcinoma cells, which explained the significant reduction of HPV16 E7 and E6*I mRNA expression. This might be due to the binding between miR-122 and suppressor of cytokine signaling 1 (SOCS1) mRNA, which is the suppressor of interferon signaling pathway. Moreover, it was identified that the miR-122 binding position was nt359-nt375 in SOCS1 mRNA. Taken together, this study indicated that HPV16 could be effectively inhibited by miR-122 through both direct binding with E6 mRNA and promoting SOCS1-dependent IFN signaling pathway. Thus, miR-122 may serve as a new therapeutic option for inhibiting HPV infection.

## Introduction

Human cervical cancer is a malignant neoplasm with the second highest female morbidity in the world [1]. Many studies revealed the close correlation between HPV infection and cervical cancer development [2]. Consequently, it is critical to inhibit HPV infection in the defense of cervical cancer.

MicroRNAs (miRNAs) can modulate gene expression post-transcriptionally [3] and function in multiple biological processes. Generally, miRNAs suppress the translation of target mRNAs by partially pairing with the 3′ un-translational region (UTR) of mRNAs or initiate mRNA degradation by completely pairing with the 3′UTR of mRNA [4]. Moreover, it is now recognized that miRNAs could also target to the coding region of critical genes and lead to transcriptional and morphological changes characteristic of differentiating mouse embryonic stem cells [5] which inspired us that miRNAs could target to HPV viral genome in host antiviral defenses.

Previous studies reported that miR-122, a liver-specific miRNA, assisted hepatitis C virus (HCV) replication by binding to its 5′UTR, resulting in stabilizing HCV genome [6]. In our previous study, miR-122 presented the anti-viral effects in Borna disease virus (BDV) persistently infected cells and hepatoma cell lines by up-regulating type I IFNs production [7], [8]. But it is still unclear whether cervical carcinoma cells, including HPV-positive and HPV-negative cells, express miR-122 and more importantly, whether miR-122 functions as anti-HPV in cervical cells.

Type I interferons (IFN-I) play critical roles in the innate immune response against viral infections. They actively participate in antiviral immunity by inducing related molecules to restrict viral proliferation and limit the spread of viral infection [9], [10]. The IFN-I bind to IFN-α/β receptor (IFNAR), which are associated with the tyrosine kinases Tyk2 and Jak1. Activated Tyk2 and Jak1 recruit and phosphorylate several STAT family members. After a complicated signal transduction, the complex formed by STAT and its recruitments translocates to the nucleus, where it binds to the upstream of IFN-stimulated response elements (ISRE) and activate the transcription of IFN-stimulated genes (ISGs), such as myxovirus resistance (Mx proteins) and 2′-5′-oligoadenylate synthetase (OAS), which function as antiviral proteins [11]. In the treatment of HPV associated human cervical cancer, IFN is one of the important agents for restriction of viral replication, which indicates the efficient antiviral effect of the IFN associated signaling pathway in HPV infection.

In this study, the constitutive expression of miR-122 were detected in different cell lines derived from human cervical cancer, which demonstrated the differential expression levels of miR-122 in those cells. Complementary bindings of miR-122 to HPV16 E6 and E7 mRNAs were predicted. The experiments, based on miR-122 over-expression or knockdown confirmed that miR-122 could directly bind with HPV16 E6 mRNA and significantly decrease the expression of E6 mRNA. Further results showed that miR-122 could pair with SOCS1, resulting in the induction of IFN-Is production, followed by down-stream response in signaling pathway. The induction of IFN inhibited HPV16 oncogenes expression. Moreover, the nt359-nt375 site of SOCS1 was identified as the binding sites of miR-122.

## Materials and Methods

### 1. Cell lines

SiHa (1–2 copies of HPV16 per cell), CaSki (about 600 copies of HPV16 per cell), HeLa (HPV18 positive) and C33A [12] (HPV negative) cell lines which derived from human cervical cancer and Huh7 cells (hepatoma cells) were cultured at 37°C in high sugar Dulbecco Modified Eagle Medium (DMEM) (Invitrogen, Carlsbad, CA) containing 5% or 10% fetal bovine serum and antibiotics (50 U/ml penicillin and 50 mg/ml streptomycin) in 5% CO_2_ incubator.

### 2. Plasmids construction and synthesis of miRNA mimics, inhibitors and mutation mimics

The expression vectors (pmCherry-E6 and pmCherry-E7) bearing HPV16 E6 and E7 gene sequences were generated by subcloning PCR products into the pmCherry-C1 expression vector (Clontech, USA). The pDC-316-EGFP-U6-miR-122 (miR-122) and pDC-316-EGFP-U6-miR-NC (miR-NC) plasmids were synthesized by Genscript (USA) as previously described [8]. The plasmid pSOCS1-EGFP was obtained from GeneChem (Shanghai, China).

The mature sequence of miR-122 (miR-122 mimic) was obtained from miR-Base database: 5′-UGGAGUGUGACAAUGGUGUUUG-3′; the sequence of miR-122 inhibitor (AMO-122) was the exact antisense copy of the mature miR-122 sequence: 5′-CAAACACCAUUGUCACACUCCA-3′; the mutant miR-122 mimics, miR-122-E6-mu and miR-122-SOCS1-mu, were designed by RNAhybrid software to eliminate the combination between miR-122 and target (HPV16 E6 and SOCS1 nt359-nt375) mRNAs, respectively. The sequences of miR-122-E6-mu and miR-122-SOCS1-mu were as follows: 5′-ACCUGACACUGUUACCACAAAC-3′, 5′-UCCUCACACACAAACCACAUUG-3′. All of the RNA oligonucleotides used in this study were synthesized and purified by GenePharma (Shanghai, China). The miR-mock and AMO-NC were irrelevant sequences supplied by GenePharma company and employed as control.

### 3. Transfection of miRNA plasmids and RNA oligonucleotides

Cells (1.5×10^5^) were seeded in 24-well plates (0.5 ml per well). After overnight culture, cells at about 70% confluency were transfected with plasmid (1 µg) or RNA oligonucleotides (30 pmol) per well by using Lipofectamine 2000 (Invitrogen, USA) as described in the instruction.

### 4. RNA extraction and RT-qPCR

The procedure details and part of primers sequences used in this study were described before [8]. The remaining primers sequences used in current study are shown in Table 1.

**Table 1:** Primers sequences list.

Gene and oligonucleotide	Sequences
HPV16 E6	
PCR forward primer	5′-GAACAGCAATACAACAAACCG-3′
PCR reverse primer	5′-CCACCGACCCCTTATATTATG-3′
HPV16 E6*I	
PCR forward primer	5′-GCAACAGTTACTGCGACGTG-3′
PCR reverse primer	5′-CAACAAGACATACATCGACCG-3′
HPV16 E7	
PCR forward primer	5′-CAGCTCAGAGGAGGAGGATG-3′
PCR reverse primer	5′-CACAACCGAAGCGTAGAGTC-3′
SOCS1	
PCR forward primer	5′- GGAACTGCTTTTTCGCCCTTA-3′
PCR reverse primer	5′- AGCAGCTCAAGAGGCAGTC-3′
MxA	
PCR forward primer	5′- GCCGGCTGTGGATATGCTA-3′
PCR reserve primer	5′-TTTATCGAAACATCTGTGAAAGCAA-3′
OAS-1	
PCR forward primer	5′-AGAAGGCAGCTCACGAAACC-3′
PCR reserve primer	5′-CCACCACCCAAGTTTCCTGTA-3′

### 5. Quantitation of mCherry expression

The fluorescent dye Hoechst 33342 (Invitrogen, USA) was added into the culture medium to stain the cell nucleus 2 h before harvest. The mCherry expression in plasmid transfected cells was detected using fluorescence microscopy (Carl Zeiss, Germany). The extracted proteins were used for mCherry fluorescence intensity measurement with a NanoDrop 3300 fluorescent spectrometer (Thermo, USA).

### 6. Western blotting

The procedure details were referred to our previous research [8]. SOCS1 antibody was obtained from Santa Cruz Biotechnology (USA). mCherry antibody was from Ythx Biotechnology (Beijing, China). Phosphorylated-STAT1 (p-STAT1) and STAT1 antibodies were purchased from Cell Signaling Technology (MA, USA) and GAPDH antibody was from ZhongShan Golden Bridge (Beijing, China).

### 7. Statistical analysis

Data were represented as means ± SD of three independent experiments. Statistical analysis was performed with Student’s t-test using SPSS 17.0. Two-tailed Pearson’s test was used to evaluate the correlation between two groups. We take *p*<0.05 as statistically significant.

## Results

### 1. Detection of constitutive expression of miR-122 in cervical carcinoma cells

**Figure 1:**
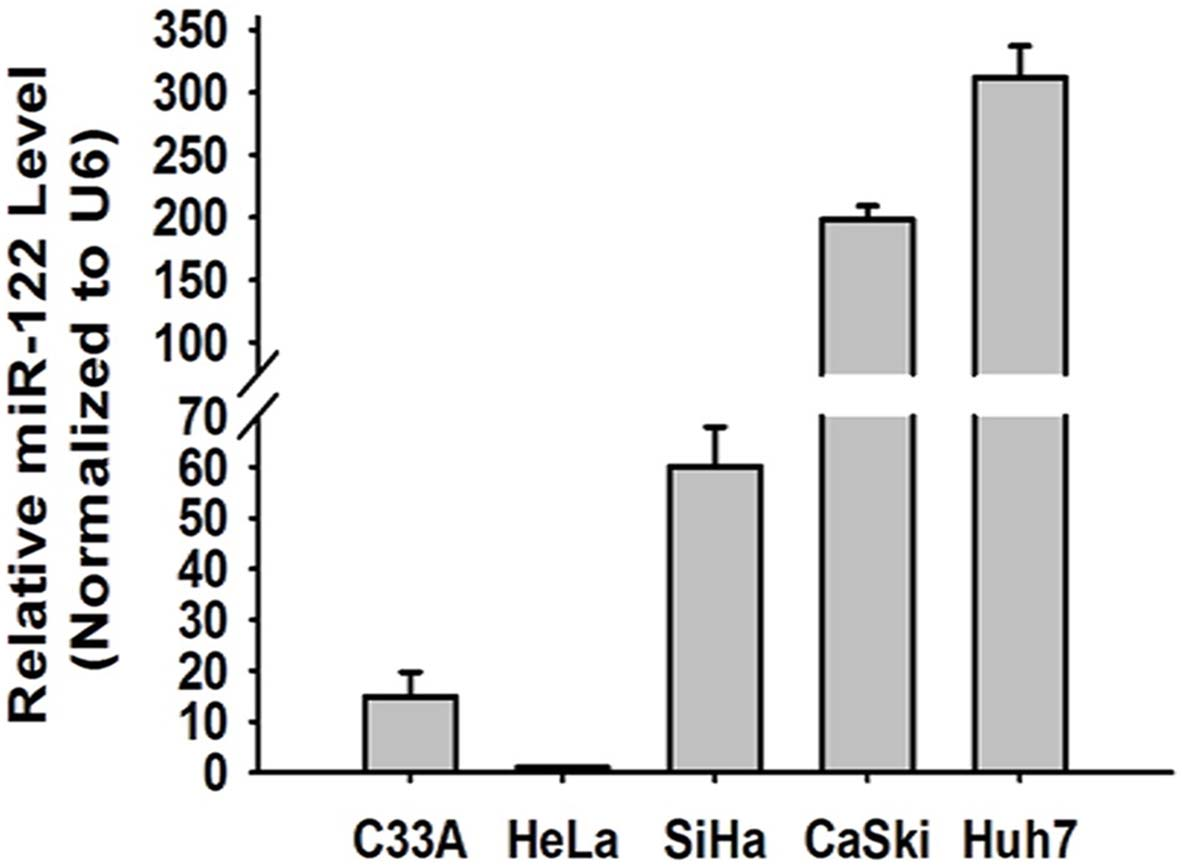
Constitutive expression of miR-122 in cervical epithelial carcinoma cells. The constitutive expression of miR-122 were separately detected in several cell lines by RT-qPCR. U6 was employed as control.

The constitutive expression of miR-122 in C33A, HeLa, SiHa and CaSki cells were detected by RT-qPCR, respectively. The miR-122 expression in Huh7 cells, human hepatoma carcinoma cells, were employed as control. The results showed that, compared with Huh7 cells, CaSki cells had the highest miR-122 expression, and HeLa cells hardly expressed miR-122. MiR-122 expression in SiHa cells was mediate, nearly one fifth of that in Huh7 cells (Figure 1). Accordingly, SiHa and HeLa cell lines were chosen for the following experiments.

### 2. MiR-122 directly inhibits HPV16 E6 oncogene

**Figure 2:**
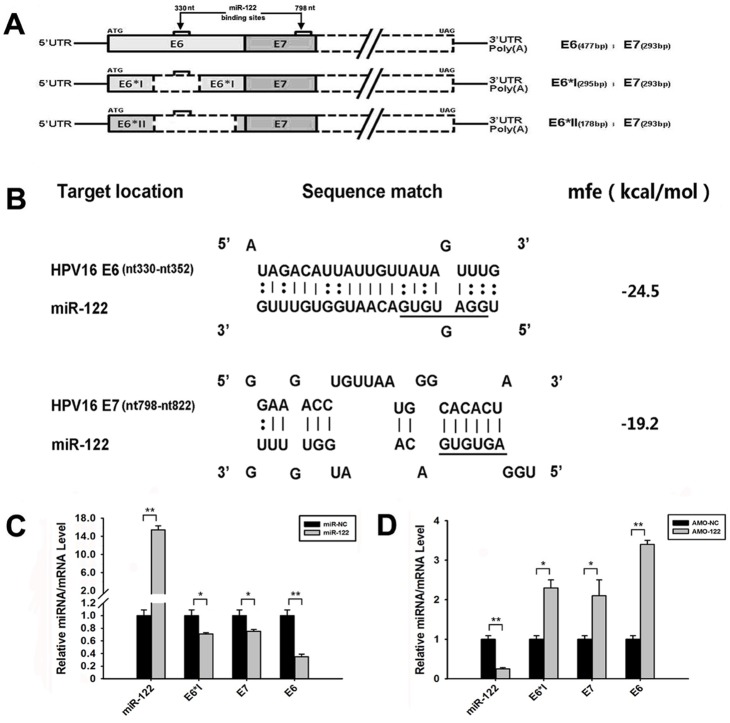
Direct inhibition of miR-122 on HPV16 E6 mRNA expression in SiHa cells. (A) The predicted binding sites of miR-122 to HPV16 E6 and E7 transcripts. (B) The predicted binding complementary sequences and binding mfe of miR-122 with the HPV16 E6 and E7 mRNAs. (C) The effects of up-regulating miR-122 on HPV16 E6*I, E7 and E6 mRNAs expression in SiHa cells, evaluated by RT-qPCR. (D) The effects of down-regulating miR-122 on HPV16 E6*I, E7 and E6 mRNAs expression in SiHa cells, evaluated by RT-qPCR. (*p<0.05, **p<0.01).

In the process of HPV16 early genes transcription, E6 oncogene has three transcriptions, E6, E6*I and E6*II mRNAs, while E7 has only one transcript. Both E6 and E7 transcripts are transcribed from the same promoter of HPV16 DNA (Figure 2A). The prediction implied that miR-122 could possibly target HPV16 E6 and E7 mRNAs, but not E6*I or E6*II mRNAs. The minimum binding free energy (mfe) [13] of these complementary bindings were shown in Figure 2B. MiR-122 plasmid and AMO-122 nucleotides were separately transfected into SiHa cells to up-regulate and block miR-122 effects, respectively. At 48 h post-transfection, the expression of miR-122, E6*I, E7 and E6 mRNAs were detected by RT-qPCR. The results showed that miR-122 down-regulated HPV16 E6 mRNA expression obviously, while it reduced E6*I and E7 mRNAs less but still significantly (Figure 2C). After blocking miR-122, the HPV16 E6 mRNA increased about 3.9-fold, while E6*I and E7 only increased about 2.3-fold and 2.1-fold (Figure 2D), respectively. These results indicated that miR-122 could directly bind with E6 mRNA.

**Figure 3:**
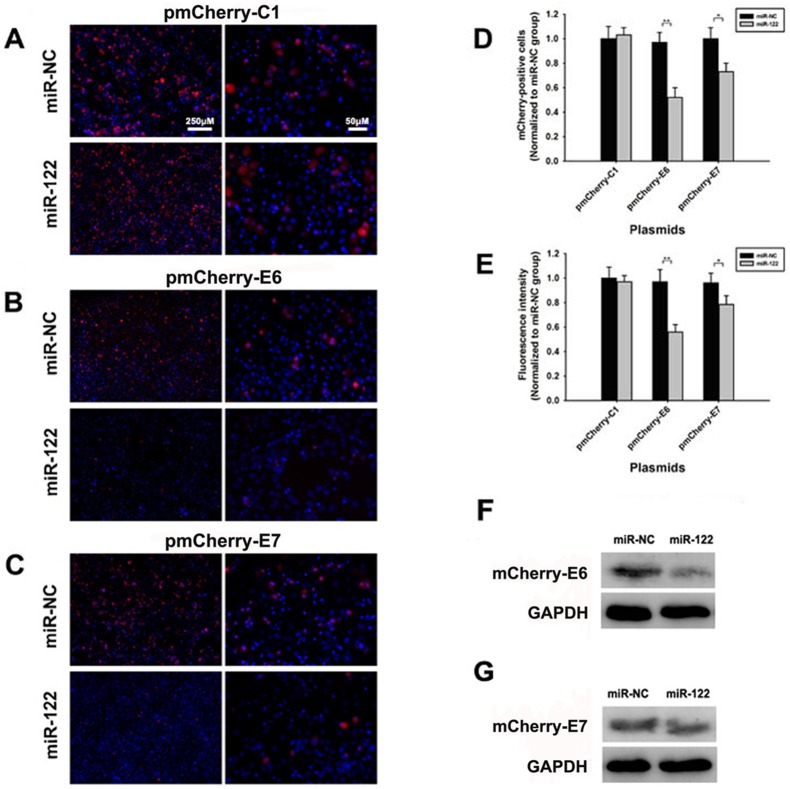
The confirmation of miR-122 direct inhibition on HPV16 E6 mRNAs. (A–C) Fluorescent images of HeLa cells co-transfected with miR-122 plasmid and pmCherry-C1, pmCherry-E6 or pmCherry-E7, respectively. (D–E) The mCherry positive cells counting and fluorescence intensity detection of HeLa cells co-transfected with miR-122 plasmid and pmCherry-C1, pmCherry-E6 or pmCherry-E7. (F–G) The mCherry-E6 and mCherry-E7 fusion proteins expression after co-transfected with miR-122 plasmid, examined by western blotting. (*p<0.05, **p<0.01).

MiR-122 plasmid was then transfected into HeLa cells with pmCherry-E6 and pmCherry-E7 plasmids to verify its inhibition effects. The relative E6 and E7 expression were measured at 36 h post-transfection by counting mCherry-positive cells, detecting mCherry fluorescence intensity and measuring the mCherry-E6 proteins expression. Compared to control group, the percentage of mCherry-positive cells decreased more obviously in miR-122 and pmCherry-E6 co-transfecting group, than that in miR-122 and pmCherry-E7 co-transfecting group (Figure 3A–D). Fluorescence intensity and western blotting results were similar as mCherry-positive cells counting results (Figure 3F and 3G). These data further indicated that miR-122 directly inhibited the expression of HPV16 E6, but not HPV16 E7. The inhibition of miR-122 on HPV16 E7 and E6*I mRNA suggested that some other pathways might play essential roles involving in the miR-122 anti-HPV effects.

**Figure 4:**
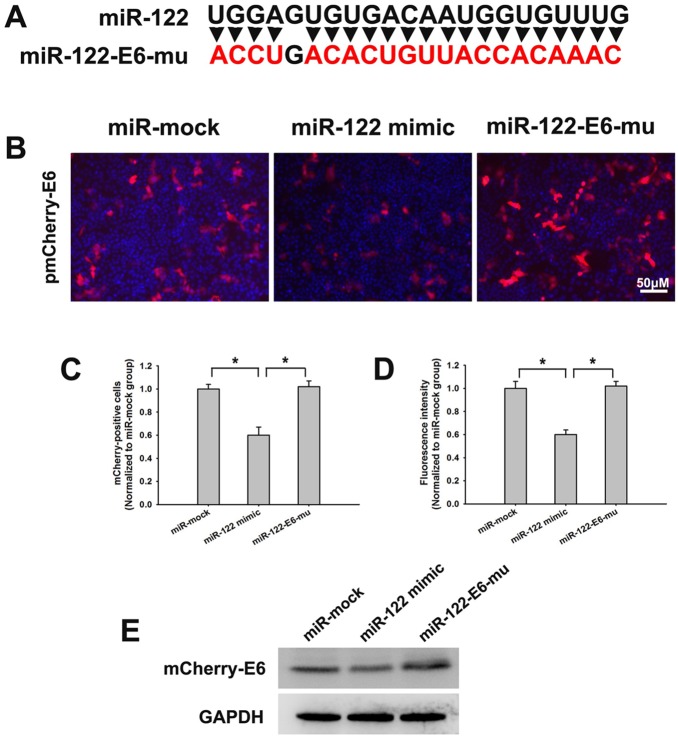
The verification of miR-122 and HPV16 E6 mRNA binding effects by mutating miR-122 sequence. (A) The nucleotide sequence miR-122-E6-mu. The red represented the mutated nucleotides. (B) Fluorescent images of HeLa cells after co-transfecting pmCherry-E6 with miR-mock, miR-122 mimic or miR-122-E6-mu, respectively. (C–D) The mCherry positive cells counting and fluorescence intensity detection of HeLa cells after co-transfecting pmCherry-E6 with miR-mock, miR-122 mimic or miR-122-E6-mu. (E) The mCherry-E6 and mCherry-E7 fusion proteins expression in HeLa cells co-transfected pmCherry-E6 with miR-mock, miR-122 mimic or miR-122-E6-mu, examined by western blotting respectively. (*p<0.05).

To confirm the binding effects of miR-122 on HPV16 E6 mRNA, pmCherry-E6 plasmid was co-transfected with miR-mock, miR-122 mimic or miR-122-E6-mu (Figure 4A) into HeLa cells, separately. According to the percentage of mCherry-positive cells and mCherry fluorescence intensity, the mCherry-E6 expression were significantly inhibited by miR-122, while it rarely affected by miR-122-E6-mu or miR-mock (Figure 4B–D). Western blotting showed the similar change as above (Figure 4E).

### 3. MiR-122 enhances type I IFN activity

**Figure 5:**
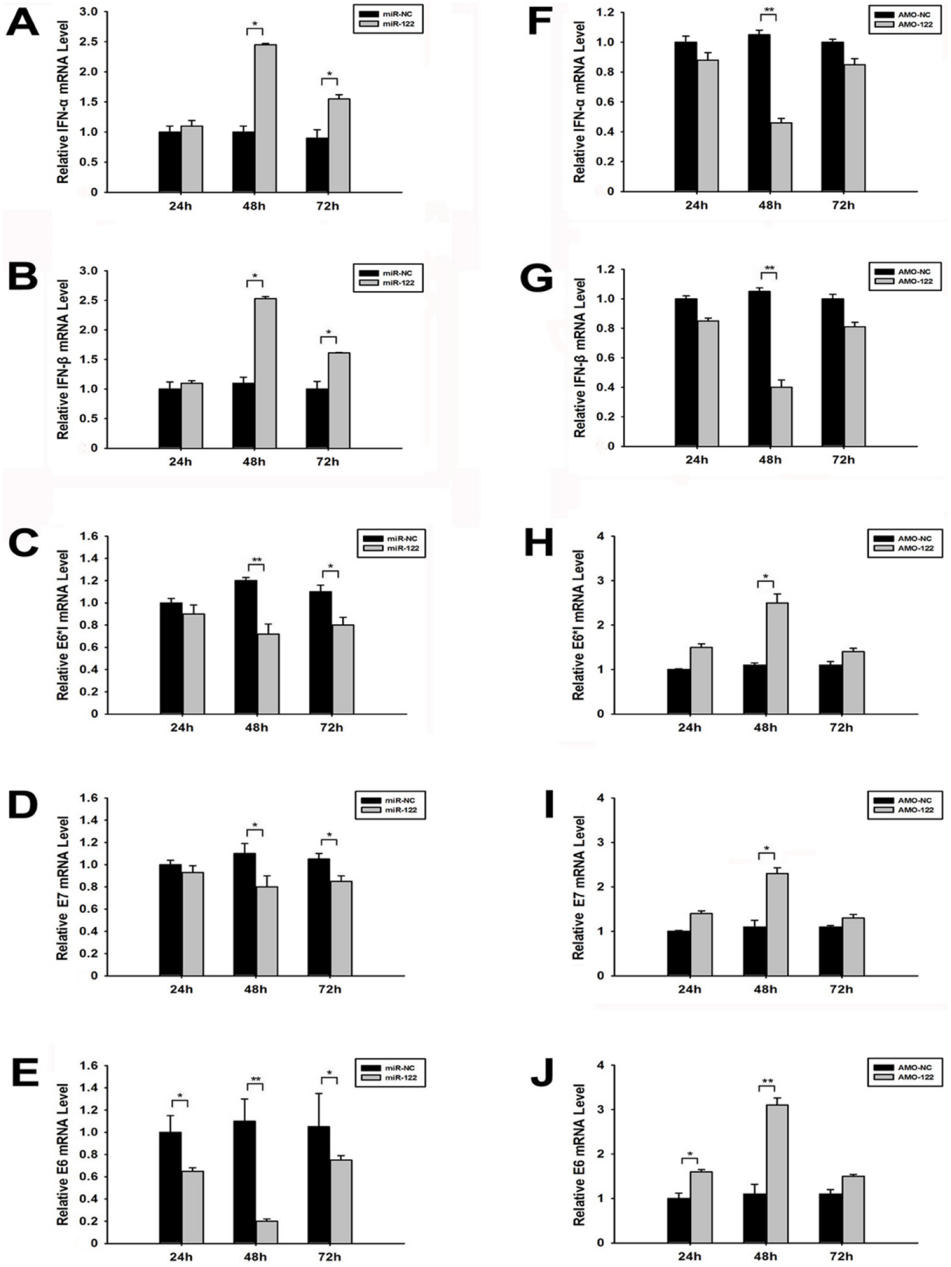
Effects of miR-122 on type I IFN, HPV16 E6*I, E7 and E6 mRNAs. (A–E) The expression of IFN-α, IFN-β, E6*I, E7 and E6 mRNAs in SiHa cells after transfected with miR-122 or miR-NC plasmid for 24 h, 48 h and 72 h, evaluated by RT-qPCR. (F–J) The expression of IFN-α, IFN-β, E6*I, E7 and E6 mRNAs after transfected with AMO-122 or AMO-NC for 24 h, 48 h and 72 h, evaluated by RT-qPCR. (*p<0.05, **p<0.01).

The inhibition of E6*I and E7 mRNAs expression by miR-122 indicated that other potential factors might be involved in miR-122 anti-HPV process. To figure it out, miR-122 plasmid and AMO-122 nucleotides were transfected into SiHa cells to detect the expression of type I IFN, E6*I, E7 and E6 mRNAs at 24 h, 48 h and 72 h post-transfection, respectively. The results showed that IFN- and IFN-β mRNAs expression levels significantly increased at 48 h post-transfection. The IFN- increased 2.3-fold and IFN-β increased 2.5-fold by miR-122 (Figure 5A and 5B), while decreased 57.2% and 61.3% by AMO-122, respectively (Figure 5F and 5G). Furthermore, at 48 h post-transfection of miR-122, there was a negative correlation between the expression of IFN-α/β and HPV16 mRNAs. We’ve also evaluated the relationship between type I IFN expression and HPV transcripts. Pearson’s correlation analysis showed a direct negative correlation between the IFN-α and IFN-β levels and HPV16 E6 mRNA expression (Figure S1). Therefore, we could conclude that the inhibition of HPV16 mRNAs by miR-122 was also attributed to the induction of type I IFN.

**Figure 6:**
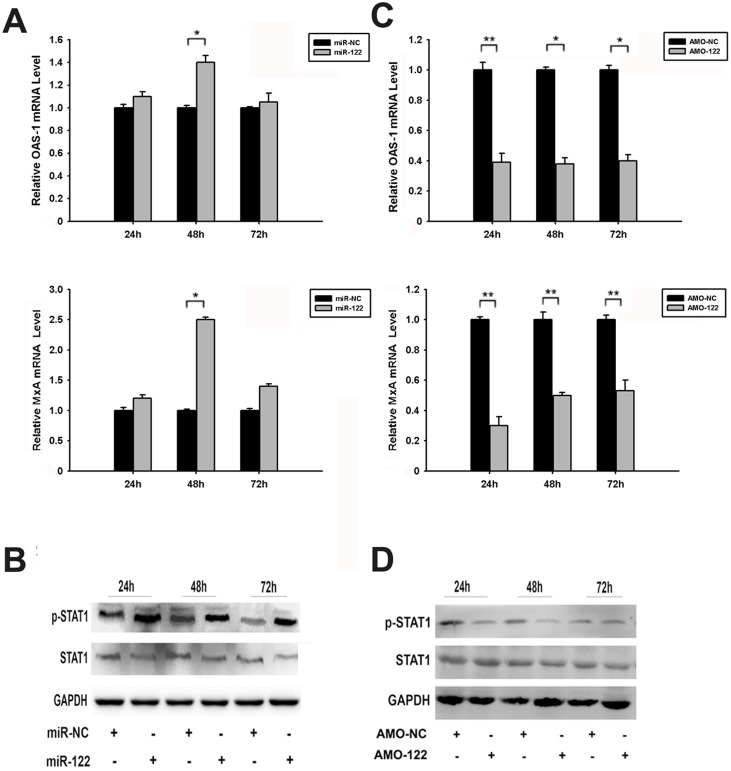
Promotion of type I IFN signal pathway by miR-122. (A) The expression of OAS-1 and MxA mRNAs in SiHa cells after transfected with miR-122 or miR-NC for 24 h, 48 h and 72 h, evaluated by RT-qPCR. (B) The phosphorylation level of STAT1 after transfected with miR-122 or miR-NC plasmid for 24 h, 48 h and 72 h, evaluated by western blotting. (C) The expression of OAS-1 and MxA mRNAs in SiHa cells after transfected with AMO-122 and AMO-NC for 24 h, 48 h and 72 h, evaluated by RT-qPCR. (D) The phosphorylation level of STAT1 after transfected with AMO-122 and AMO-NC for 24 h, 48 h and 72 h, evaluated by western blotting. (*p<0.05, **p<0.01).

To verify the type I IFN signal pathway has been promoted by miR-122 in cervical carcinoma cells, IFN stimulated genes and the activity of STAT proteins were detected respectively. As expected, the expression of OAS-1 and MxA’s mRNAs increased after miR-122 mimic transfection, and the significant increase exhibited at 48 h post-transfection (Figure 6A). The phosphorylation levels of STAT1 protein increased clearly enough to reflect the same tendency after treated SiHa cells with miR-122 mimic (Figure 6B). Loss function of miR-122 decreased the expression of OAS-1 and MxA’s mRNAs (Figure 6C). The phosphorylation levels of STAT1 protein reduced by AMO-122, either (Figure 6D).

### 4. MiR-122 increases type I IFN expression through blocking SOCS1

**Figure 7:**
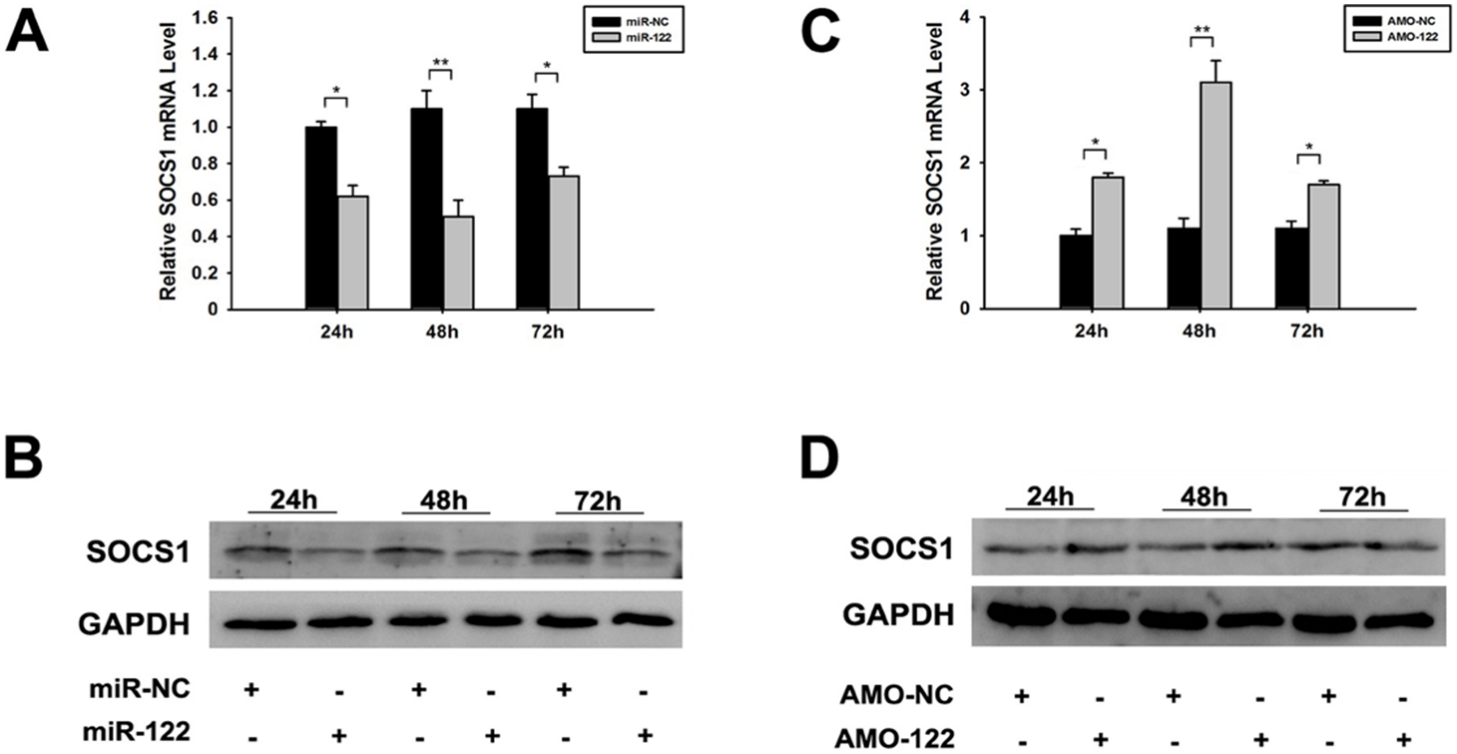
Inhibition effects of miR-122 on SOCS1 mRNA. (A–B) Inhibition of SOCS1 by transfecting miR-122 in SiHa cells, detected by RT-qPCR and western blotting at 24 h, 48 h and 72 h separately. (C–D) Up-regulation of SOCS1 by transfecting AMO-122 in SiHa cells at 24 h, 48 h and 72 h separately. (*p<0.05, **p<0.01).

Our previous study showed that SOCS1 effectively regulated type I IFN signal pathway and miR-122 could regulate the SOCS1 protein expression in Huh7 cells [7]. In this study, miR-122 plasmid and AMO-122 nucleotide were separately transfected into SiHa cells, and the expression of SOCS1 was evaluated at 24 h, 48 h and 72 h post-transfection. The results showed that both mRNA and protein levels of SOCS1 decreased, especially at 48 h post-transfection of miR-122 (Figure 7A and 7B). As expected, SOCS1 increased significantly after transfected AMO-122 (Figure 7C and 7D). As SOCS1 acted as the inhibitor of type I IFN, the results indicated that miR-122 affected the expression of type I IFN signal pathway by inhibiting SOCS1.

**Figure 8:**
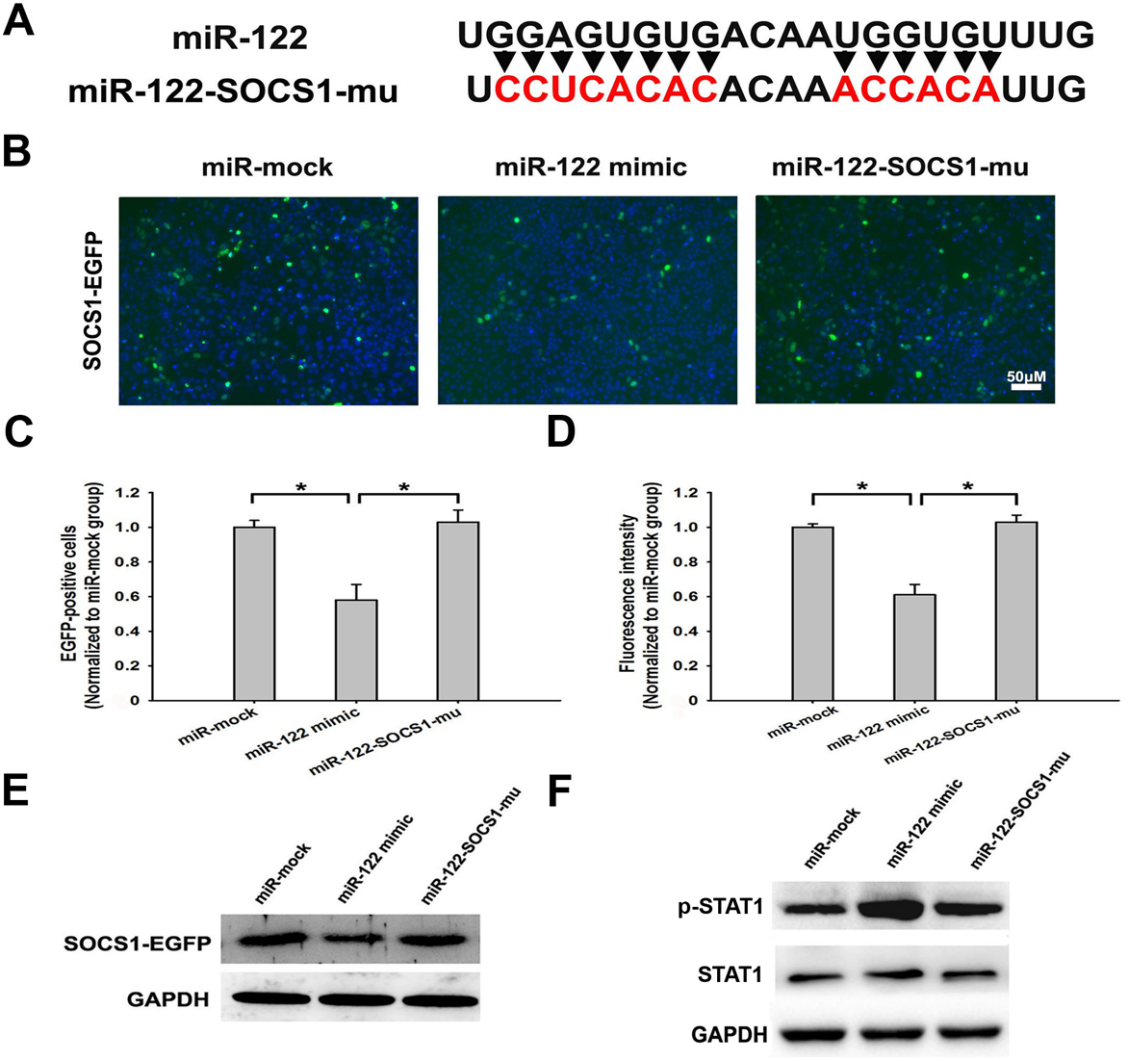
The verification of miR-122 binding site in SOCS1. (A) The nucleotide sequence of miR-122-SOCS1-mu. The red represented the mutated nucleotides. (B) Fluorescent images of HeLa cells co-transfecting pSOCS1-EGFP plasmid with miR-mock, miR-122 mimic or miR-122-SOCS1-mu, respectively. (C–D) The SOCS1-EGFP positive cells counting and fluorescence intensity detection of HeLa cells after co-transfected pSOCS1-EGFP plasmid with miR-mock, miR-122 mimic or miR-122-SOCS1-mu. (E) The SOCS1-EGFP protein expression in HeLa cells co-transfecting pSOCS1-EGFP plasmid with miR-mock, miR-122 mimic or miR-122-SOCS1-mu, detected by western blotting. (F) The phosphorylation level of STAT1 in SiHa cells after transfected miR-mock, miR-122 mimic or miR-122-SOCS1-mu, detected by western blotting. (*p<0.05).

The previous study also indicated that nt359-nt375 of SOCS1 might be the target of miR-122 in Huh7 cells [7]. To confirm the binding effects of miR-122 on SOCS1 mRNA in HeLa cells, pSOCS1-EGFP expressing plasmid was co-transfected with miR-mock, miR-122 mimic or miR-122-SOCS1-mu (Figure 8A), separately. Compared with miR-mock transfected cells, SOCS1-EGFP expression was inhibited obviously by miR-122, but not miR-122-SOCS1-mu (Figure 8B). MiR-122-SOCS1-mu had the similar effect on SOCS1-EGFP as miR-mock (Figure 8C and 8D). Western blotting results showed the same change of SOCS1-EGFP expression as above (Figure 8E), which indicated that miR-122-SOCS1-mu did eliminate the binding effects of miR-122 on SOCS1 mRNA. Furthermore, to confirm the response to IFN-I pathway after blocking SOCS1 by miR-122, the expression of STAT1 was detected by western blotting after separately transfected miR-122 mimic and miR-122-SOCS1-mu into SiHa cells. As shown, the phosphorylation level of STAT1 increased obviously by miR-122 mimic, instead of miR-mock or miR-122-SOCS1-mu (Figure 8F).

## Discussion

It has been reported that miRNAs could act as many viruses’ suppressors, including both RNA viruses and DNA viruses. Several miRNAs (miR-29a, miR-29b, miR-149, miR-378, miR-324-5p) targeted to the human immunodeficiency virus (HIV) genome and inhibited viral infection in T cells [14]. MiR-507 and miR-136 could restrict the virus replication by targeting the genes of influenza virus Polymerase B2 (PB2) and Hemagglutinin (HA), respectively [15]. MiR-125a-5p, miR-199a-3p and miR-210 have been reported to directly suppress HBV expression [16], [17], [18]. Yet, the interaction between miR-122 and HPV has not been studied before.

In our study, the antiviral capability of miR-122 on HPV 16 was verified. Also, the study showed that the constitutive expression of miR-122 in several human cervical carcinoma cell lines were different and some cervical cells have higher miR-122 expression. Furthermore, based on the prediction, we found that miR-122 could complementally bind to HPV16 E6 and E7 mRNAs rather than HPV16 E6*I mRNA. According to predicted mfe, the combining ability of miR-122 to HPV16 E6 mRNA is better than to E7 mRNA (Figure 2B). Up-expression and down-expression of miR-122 verified that miR-122 could only directly bind with HPV16 E6 mRNA and decrease its level greatly. This significant inhibition of E7 and E6*I mRNAs suggested that miR-122 might also induce some other cellular antiviral signal pathways to influence HPV’s mRNAs expression.

Type I IFN was reported to effectively suppress HBV replication in vivo [19], and it was also involved in inhibiting HPV E6 or E7 proteins and affecting the cell cycle [20]. We previously demonstrated that over-expression of miR-122 induced the production of type I IFN in BDV persistent infected OL cells [8]. Accordingly, type I IFN became our first hypothesis of miR-122 antiviral pathway.

The type I IFN signal pathway is a complicated network which involves many critical molecules. MxA and OAS-1 are two effective antiviral proteins in the type I IFN pathway. MxA was reported to inhibit a wide range of RNA viruses, including influenza viruses and members of bunyavirus family at an early stage in their life cycle, soon after viral entry into host cells and before viral genome amplification [21]. OAS, works as a crucial enzyme for the degradation of RNA [22] and was recently reported to inhibit hepatitis B virus replication [23]. After transfected miR-122 into SiHa cells, type I IFN and associated ISGs increased significantly, indicating that miR-122 might also anti-HPV by inducing IFN-I pathway.

Through prediction, the combination of miR-122 and SOCS1 was identified, which inspired us that miR-122 might induce IFN-I pathway by blocking the negative regulator of it. The following experiments confirmed our hypothesis and furthermore, identified the binding sites at nt359-nt375 in SOCS1 mRNA.

The main cell line used in this study was SiHa, because it carries HPV16, the major infection type, and has middle level miR-122 constitutive expression (Figure 1). In Figure 3, Figure 4 and Figure 8 (except Figure 8F), the experiments were presented in HeLa cells. This was because HeLa cells expressed little endogenous miR-122, which made the antiviral effects of miR-122 more obvious shown in the fluorescent images by transfecting exogenous miR-122 plasmid. As we co-treated HeLa cells with miR-122 and HPV16 E6 plasmids, the HPV18 oncogenes in HeLa cells did not influence our conclusion that miR-122 could act as anti-HPV reagent and might serve as a therapeutic option in human cervical cancer.

In conclusion, this study showed that miR-122 inhibited HPV through both binding to E6 mRNA directly and promoting type I IFN signaling pathway indirectly in cervical carcinoma cell lines and might be a potentially viable therapeutic option for HPV infection.

## Supporting Information

Figure S1 **The correlation analysis between HPV16 E6 and type I IFN.** (A) Pearson correlation analysis for the HPV16 E6 mRNA expression and IFN-α levels. (r = 0.911, *p*<0.001) (B) Pearson correlation analysis for the HPV16 E6 mRNA expression and IFN-β levels. (r = 0.909, *p*<0.001).
(DOCX)
