# Supplementary figures and images for: MiR-122 Directly Inhibits Human Papillomavirus E6 Gene and Enhances Interferon Signaling through Blocking Suppressor of Cytokine Signaling 1 in SiHa Cells
Source: PLoS One. 2014 Sep 29;9(9):e108410. doi: 10.1371/journal.pone.0108410 (PMC4180754; doi:10.1371/journal.pone.0108410)

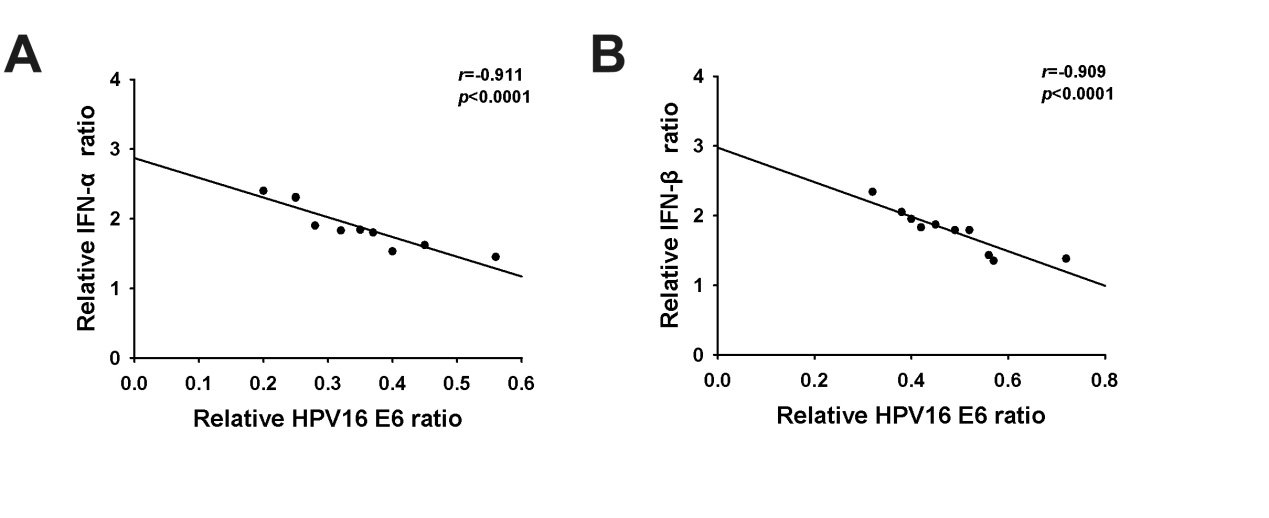


**Figure S1. The correlation analysis between HPV16 E6 and type I IFN.**

Supplement: Figure S1 — The correlation analysis between HPV16 E6 and type I IFN. (A) Pearson correlation analysis for the HPV16 E6 mRNA expression and IFN-α levels. (r = 0.911, p<0.001) (B) Pearson correlation analysis for the HPV16 E6 mRNA expression and IFN-β levels. (r = 0.909, p<0.001). (DOCX) [file pone.0108410.s001.docx]
